# An Experimentally Evaluated Thermodynamic Approach to Estimate Growth of Photoheterotrophic Purple Non-sulfur Bacteria

**DOI:** 10.3389/fmicb.2020.540378

**Published:** 2020-09-03

**Authors:** Anna Doloman, Lance C. Seefeldt

**Affiliations:** Department of Chemistry and Biochemistry, Utah State University, Logan, UT, United States

**Keywords:** thermodynamics, photoheterotrophic growth, *Rhodopseudomonas palustris* nifA^∗^, photobioreactor, hydrogen gas, nitrogen fixation

## Abstract

Distribution of energy during the growth and formation of useful chemicals by microorganisms can define the overall performance of a biotechnological system. However, to date, this distribution has not been used to reliably predict growth characteristics of phototrophic microorganisms. The presented research addresses this application by estimating the photon-associated Gibbs energy delivered for the photoheterotrophic growth of purple non-sulfur bacteria and production of dihydrogen. The approach is successfully evaluated with the data from a fed-batch growth of *Rhodopseudomonas palustris* nifA^∗^ fixing N_2_ gas in phototrophic conditions and a reliable prediction of growth characteristics is demonstrated. Additionally, literature-available experimental data is collected and used for evaluation of the presented thermodynamic approach to predict photoheterotrophic growth yields. A proposed thermodynamic framework with modification to account for the phototrophic growth can be used to predict growth rates in broader environmental niches and to assess the possibility for the development of novel biotechnological applications in light-induced biological systems.

## Introduction

In the era of extensive computational research and increasing availability of omics tools, increasingly complex models for microbial transformations have been proposed. With the ability to model complex systems, simpler models are either disregarded or considered to not have enough accuracy to describe a specific biological transformation. However, in the case where an in-depth enzymatic level of accuracy is not needed or otherwise not possible, simplified models may be valuable. Simplified models can have particular value if the evaluated system is of biotechnological importance and a stoichiometrically reliable analysis of microbial activity is needed. A stoichiometrically correct prediction of microbial transformations in simplified models allows accurate prediction of the rates of substrate consumption and product formation, as well as total generation of biomass. Such prediction is a necessary and powerful tool for bioprocess engineers and laboratories investigating microbial bioconversions pathways with an aim toward a complete and controlled resource recovery.

Modelsthat allow for reliable prediction of microbial transformations and product yields arebased on the thermodynamics governing the behavior of energy transducing systems (Doelle, 1969; Nicholls and Ferguson, 2002). It is a true “black-box” model where no gene-protein relationships are considered, but electron and energy balances from oxidation-reduction reactions control the transformations in a biological system. Contrary to the commonly used Monod, Contois, Teissier and other simplified kinetic models describing bacterial growth (Kargi and Shuler, 1979), a thermodynamic approach to modeling does not account for enzymatic activity and does not require any prior experimental knowledge of the studied system, except for the knowledge of the environmental parameters (types of electron donors and acceptors, sources of nitrogen). The biochemical conversions in microbial systems produce changes in the Gibbs energy and all the energy available for the system is distributed between the catabolic and anabolic reactions, influencing the bioconversion stoichiometries. This concept was first introduced by McCarty (McCarty, 1965; Christensen and McCarthy, 1975) and later evaluated by a number of researchers (Heijnen et al., 1992; VanBriesen, 2002; Kleerebezem and Van Loosdrecht, 2010; Araujo et al., 2016; Brock, 2019), comparing the framework with other models describing bacterial growth. The thermodynamic framework has been successfully applied to predict the performance of various heterotrophic bacteria (McFarland and Sims, 1991; Heijnen, 1994) and is considered a reliable evaluation tool for bioprocess design (Tchobanoglous et al., 2014). However, to date, there is no literature reporting simple and reliable application of this thermodynamic framework to evaluate phototrophic microbial systems (von Stockar et al., 2006). An approach closest in application was described by von Stockar et al. (2011) and relies on calorimetric measurements of the heat transmission coefficients in microbial systems to make phototrophic growth predictions. Despite usefulness of such an approach, application would require multiple instrumental measurements tailored for a growth system and organism of interest and cannot be generalized. For a ground-level assessment of a biological system, a more generalized approach is needed that doesn’t require intensive instrumental analysis and incorporates photon-associated energy into the oxidation-reduction system of equations. Some authors modeled light-induced transfer of electrons in the bacterial photosynthetic system and its influence on the bacterial growth rates, relying on experimental rates for photon absorption in light-harvesting complexes and correspondent concentrations of reaction centers/bacteriochlorophylls (Klamt et al., 2008). Others have focused on mathematical derivations of photon chemical potential for non-equilibrium thermodynamics (Meszéna and Westerhoff, 1999). However, neither of those derivations are directly applicable to the established thermodynamic framework by McCarty (Rittmann and McCarty, 2001).

Here we present a simplified approach to incorporate the photon-associated energy in units compatible with an existing thermodynamic framework. We also validate predicted phototrophic bioconversion rates with an experimental dataset: a 3L laboratory photobioreactor was set up to grow purple non-sulfur bacteria and growth was evaluated in the thermodynamic framework. Finally, we calculate bacterial growth yields for multiple literature-reported phototrophic scenarios and compare them with reported yield values.

## Materials and Methods

### Experimental

A strain of purple non-sulfur bacteria, *Rhodopseudomonas palustris* nifA^∗^ (McKinlay and Harwood, 2010) was grown in a defined mineral medium containing Na_2_HPO_4_ (1.77 g/L), KH_2_PO_4_ (1.77 g/L), 0.1% (v/v) concentrated base solution, 0.5% (v/v) Wolfe’s vitamins solution and 30 mM sodium acetate, as a carbon source. Concentrated base solution consisted of (g/L): nitrilotriacetic acid (20), MgSO_4_ (28.9), CaCl_2_.2H_2_O (6.67), (NH_4_)_6_Mo_7_O_24_.4H_2_O (0.0185), FeSO_4_.7H_2_O (0.198) and 10%(v/v) metals solution, prepared separately (g/L): EDTA (2.5), ZnSO_4_.7H_2_O(10.95), FeSO_4_.7H_2_O (5), MnSO_4_.H_2_O (1.54), CuSO_4_.5H_2_O (0.392), Co(NO_3_)_2_.6H_2_O (0.25), Na_2_B_4_O_7_.10H_2_O (0.177). The Wolfe’s vitamin solution contained (g/L): para-aminobenzoic acid (0.005), folic acid (0.002), lipoic acid (0.005), riboflavin (0.005), thiamine (0.005), nicotinic acid (0.005), pyridoxamine (0.01), pantothenic acid (0.005), cobalamin (0.0001), biotin (0.002). The pH of the media was adjusted to pH 6.9-7.1. For initial inoculum, *R. palustris* was grown anaerobically in 120 ml serum vials (60 ml liquid volume) and media was flushed with N_2_ gas for 30 min prior to being autoclaved at 121°C for 20 min. For bioreactor growth experiments, the media was autoclaved first, aseptically pumped into the vessel, and flushed with N_2_:CO_2_ (80:20) mixture for 2 h. Cultures were grown at 30 ± 1°C with 60 W incandescent light (spectrum provided on Supplementary Figure S1) (serum vials) or with 3 × 6 fluorescent lamps, 17 W each (spectrum provided on Supplementary Figure S2), delivering a constant photon flux of 340 μmol × m^–2^ × s^–1^ (wavelength of 340–1040 nm).

The 3L photobioreactor was inoculated with 5% (v/v) of late-exponential phase grown *R. palustris* nifA^∗^ culture and operated in a fed-batch mode for 3 weeks. The pressure inside the reactor was kept at 1.06 atm (101.325 kPa), with P_*CO2*_ = 0.21atm (21.28 kPa) and P_*N2*_ = 0.95 atm (96.26 kPa). The gas outlet was constantly kept open throughout the operation to eliminate pressure build-up due to the production of H_2_ gas. The bioreactor was aseptically sampled to determine cell dry weight, acetate concentration, and headspace hydrogen gas generation. Gas composition was determined using a Shimadzu GC-8A gas chromatograph (Shimadzu Scientific Instruments, Inc.)with an in-line thermal conductivity detector. Argon was used as a carrier gas (135 kPa), the temperature of the injector and detector was set to 100°C, and the column was kept at 60°C. Injection volumes were 200 μl. A series of standards with known concentrations of H_2_ balanced with N_2_ was used to prepare a standard curve to evaluate changes in the bioreactor headspace gas composition. Acetate concentration was determined by analyzing the culture supernatant using H-NMR (Bruker, San Jose, CA), using D_2_O as frequency lock for the machine and maleic acid (20 mM) as an internal standard. TopSpin 3.6.2 was used to analyze the NMR data.

### Theoretical

The thermodynamic approach for predicting bacterial growth yield is based on the changes in Gibbs energy from metabolic oxidation-reduction reactions and is used here as originally described by McCarty (McCarty, 1965; Tchobanoglous et al., 2014), with a novel modification to account for the photon-associated energy. Oxidation-reduction reactions describe electron flow between electron-donor (often also the carbon source) and electron-acceptor (CO_2_, N_2_, H^+^, O_2_, etc.). The existing framework relies on the following assumptions (Rittmann and McCarty, 2001): (1) bacterial cells only capture 55% of the available energy associated with oxidation-reduction reactions. This assumption comes from the knowledge that microorganisms have in general a 45–65% efficiency of energy transfer from catabolic reactions (oxidation of substrate) to the formation of ATP, which is considered as a main energy currency in the cell (Thauer et al., 1977; McCarty, 2007); (2) ammonium is the only nitrogen source that does not undergo additional chemical conversions in the cell, instead being directly incorporated into the cellular amino acids and proteins; use of other nitrogen sources (NO_2_^–^, NO_3_^–^, N_2_) is “penalized” by subtracting the associated energy required to convert them into ammonium; and (3) pyruvate is a universal biosynthetic intermediate for cellular conversions, and the energy required to convert 1 e^–^ eq of pyruvate into 1 e^–^ eq of cells is subtracted from the total energy acquired in redox reactions. In the proposed here modifications, photon-associated energy and the energy produced due to the oxidation-reduction reactions sum to the total energy available for cellular processes.

Oxidation-reduction reactions are written in a form of half-reactions per electron equivalent (1 e^–^ eq). Thus, photon-associated energy needs to be also expressed in the electron equivalent. In anoxygenic bacterial photosynthesis (specifically, purple non-sulfur bacteria), energy from one photon (800 nm) in the form of electromagnetic radiation is absorbed by a molecule of bacteriochlorophyll (a photosynthetic pigment) (Fleming and Van Grondellet, 1997). Absorbed energy is transferred between pigments in two light-harvesting complexes (LH2 to LH1) and then to the reaction center (RC), where RC-associated bacteriochlorophylls receive the energy equivalent of a photon with 870 nm wavelength (Hoff and Deisenhofer, 1997). This photon energy is used in the quinone reaction cycle at the RC, where the photoactivated cytochrome donates one electron for reduction of quinol (in total two photons and two electrons are needed to completely reduce quinol) (Okamura et al., 2000). Therefore, for the purpose of modeling, 1 mol of photons can be thought of as equivalent to 1 mol of immobilized electrons, if the bacterial LHCs are not limited by light. This assumption allows us to express photon-associated energy in a suitable format for the thermodynamic framework using the following formula (Nicholls and Ferguson, 2003):

(1) $$△\,G_{p\,h\,o\,t\,o\,n}=N_{A}\times h\times \nu =\frac{N_{A}\times h\times c}{\lambda }$$

Where, *N_A* is Avogadro number 6.022 × 10^23^ mol^–1^; *h* is a Planck constant 6.62 × 10^–34^ J × s; *c* is a speed of light 3 × 10^8^ m × s^–1^; ν is a frequency of the radiation, s^–1^; λ is the wavelength of light, nm. Since the estimated △*G*_*photon*_ serves as a source of energy for cellular metabolic processes, it must be used with a negative sign for further thermodynamic calculations. The 870 nm wavelength of the light used in the proposed framework is specifically descriptive of the photosystem of anaerobic purple non-sulfur bacteria. Other anaerobic photosynthetic bacteria (green sulfur/non-sulfur bacteria and anaerobic cyanobacteria) absorb photons with different wavelengths and that should be considered if the framework is to be used for correspondent analyses.

## Results and Discussion

This study presents an addition to the well-established thermodynamic framework to analyze microbial growth. The original thermodynamic framework was proposed by McCarty (McCarty, 1965) and we suggest a modification to account for the photon-associated energy available for bacterial growth. An example calculation of theoretical growth yield is based on the scenario for anaerobic photoheterotrophic growth of purple non-sulfur bacteria in a minimal media (with N_2_ and acetate as nitrogen and carbon sources). The evaluated system is regarded to have no light limitations, and that photosystem of the purple non-sulfur bacteria is not limited by the photons. Table 1 summarizes the half-reactions and associated free energies available to drive the bioconversion in the evaluated system.

**TABLE 1:** The half-reactions and associated free energies describing diazotrophic anoxic photoheterotrophic bacterial growth.

**Half-reactions and energy contributing sources**	**Standard Gibbs energy (pH = 7; t° = 25°C),Δ*G*^0^, *kJ*/*moleeq*^1^**	**References**
***Oxidation of acetate***	−27.68	Tchobanoglous et al., 2014
$\frac{1}{8}\,C\,H_{3}\,C\,O\,O^{-}+\frac{3}{8}\,H_{2}\,O\to \frac{1}{8}\,C\,O_{2}+\frac{1}{8}\,H\,C\,O_{3}^{-}+H^{+}+e^{-}$		
***Reduction of nitrogen gas***		Tchobanoglous et al., 2014
$\frac{1}{6}\,N_{2}+\frac{4}{3}\,H^{+}+e^{-}\to \frac{1}{3}\,N\,H_{4}^{+}$	27.47	
$H^{+}+e^{-}\to \frac{1}{2}\,H_{2}$	40.46	
**Light energy** (870 nm), △*G*_*photon*,870*nm*_	−137	Current study
**Overall energy capturing reaction:**		
$\frac{1}{8}\,C\,H_{3}\,C\,O\,O^{-}+\frac{3}{8}\,H_{2}\,O+\frac{1}{8}\,N_{2}+\frac{1}{4}\,H^{+}+1\,h\,\nu \to \frac{1}{8}\,C\,O_{2}+\frac{1}{8}\,H\,C\,O_{3}^{-}+\frac{1}{4}\,N\,H_{4}^{+}+\frac{1}{8}\,H_{2}$		
$△\,G_{R}=\left(-27.68\right)+\left(\frac{3}{4}\times 27.47\right)+\left(\frac{1}{4}\times 40.46\right)+\left(-137\right)=-133.96\,k\,J/m\,o\,l\,e\,e\,q$		

*^1^“eeq” is electron equivalent. Refers to the one-electron reduction reactions.*

In Table 1, the obtained energy for bacterial growth, △*G*_*R*_, is distributed between catabolic and anabolic cellular processes (see detailed calculations in Supplementary Material) and is used to calculate a case-specific stoichiometry of biological reaction (Rittmann and McCarty, 2001):

(2) $$R=f_{e}\times R_{a}+f_{s}\times R_{c\,s}-R_{d}$$

Where, *R* is an overall balanced reaction; *f_e* is the fraction of electron donor used for energy; *R_a* is a half-reaction for electron acceptor; *f_s*is the fraction of electron donor used for cell synthesis; *R*_*cs*_ is a half-reaction for synthesis of cell mass; *R*_*d*_ is a half-reaction for electron donor. The resulting balanced equation is:

$$R=0.125\,C\,H_{3}\,C\,O\,O^{-}+0.0740\,N_{2}+0.127\,H^{+}+0.204\,H_{2}\,O$$

$$+1\,h\,\nu \to 0.0214\,C_{5}\,H_{7}\,O_{2}\,N+0.018\,C\,O_{2}+0.125\,H\,C\,O_{3}^{-}$$

(3) $$+0.127\,N\,H_{4}+0.0634\,H_{2}$$

Where C_5_H_7_O_2_N is a general formula describing bacterial biomass composition (MW = 113 g/mol).

The resulting stoichiometrically balanced equation for the photoheterotrophic growth of purple non-sulfur bacteria grown with N_2_ as a source of nitrogen and acetate as a carbon source can be used to calculate growth yields (*Y*_*X*/*S*_) and product yields (*Y*_*P*/*S*_) per substrate consumed (S). Hydrogen gas is used as an example product in this study. The yield of biomass on acetate, *Y*_*X*/*Acetate*_: (0.0214 mol × 113 g × mol^–1^)/(0.125 mol × 59 g × mol^–1^) = 0.328 g cells/g acetate; Yield of hydrogen gas on acetate,*Y*_*H*_2_/*Acetate*_: (0.063 mol × 23.455L × mol^–1^)/(0.125 mol × 59 g × mol^–1^) = 0.2 L H_2_/g acetate.

**FIGURE 1:**
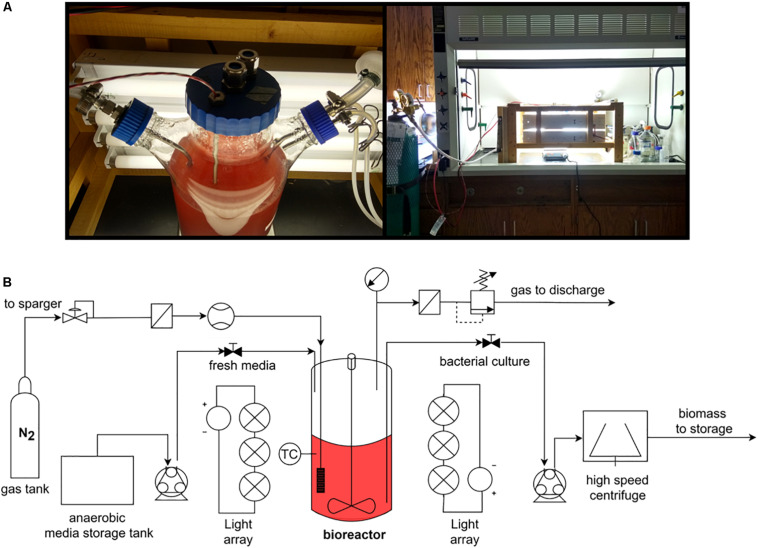
The 3L photobioreactor set-up **(A)** and an operational flow chart **(B)**.

To test the accuracy of the proposed thermodynamic framework, a photobioreactor was set up with purple non-sulfur bacterial strain *Rhodopseudomonas palustris* nifA^∗^. The strain is a stable mutant with a 48 bp deletion in a *nifA* gene, which has a constitutively expressed nitrogenase and a dysfunctional uptake hydrogenase (McKinlay and Harwood, 2010). *R. palustris* nifA^∗^ was grown in batch serum vials and fed-batch photobioreactor (PBR) trials to compare the yield characteristics in two different environments. The photobioreactor set-up and an overall flow chart are provided in Figure 1. The choice of a nifA^∗^ mutant stain of *R. palustris* allowed detecting stoichiometric hydrogen production. Since this mutant has a dysfunctional uptake hydrogenase and thus cannot re-uptake hydrogen, potential errors in product yield calculations are minimized. The observed and theoretical calculated *R. palustris* nifA^∗^ growth characteristics are provided in Table 2.

**TABLE 2:** Observed and theoretical calculated growth characteristics of *R. palustris* nifA^∗^ grown anaerobically in the presence of light with acetate as a carbon source.

**Test #**	**Increase in dry weight, g/L**	**Growth rate^1^, μ_*net*_, h^–1^**	**Hydrogen yield, *Y*_*H*_2_/*Acetate*_, mol/mol**	**Biomass yield, *Y*_*X*/*Acetate*_, mol/mol**	**Biomass yield, *Y*_*X*/*Acetate*_, g/g**
Predicted values from thermodynamic framework	n/a	n/a	0.5^2^	0.17	0.33
Batch 1	0.2904	0.1	0.013	0.128	0.176
Batch 2	0.3473	0.028	0.022	0.128	0.177
Batch 3	0.391	0.084	0.154	0.162	0.224
PBR 1	0.3727	0.023	0.39	0.1828	0.248
PBR 2	0.192	0.025	0.48	0.1669	0.28

*“PBR” – photobioreactor. ^1^Growth rate, $\mu_{n\,e\,t}=\frac{\ln X_{2}-l\,n\,X_{1}}{t_{2}-t_{1}}$, where *X_1* and *X_2* are cell concentrations during the exponential growth phase at times *t_1* and *t_2*; ^2^Yield of hydrogen gas was recalculated for the experimental conditions: 30°C (303K) and 1.06 atm (107.4 kPa).*

As can be seen from the Table 2 data, PBR growth trials produced growth yield values closer to the predicted values in the thermodynamic framework. Even though the growth rates (μ, h^–1^) were higher in batch trials, hydrogen production yields were significantly lower when compared to the PBR trials. A possible explanation is in the PBR design: a one-way valve attached to the gas outlet allowed for a continuous discharge of the generated hydrogen, thus minimizing self-inhibition. Batch cultures, however, were not relieved of the accumulated hydrogen, thus allowing self-inhibition effects over the course of cultivation. A note on the batch 3 data from Table 2: this batch had a higher starting biomass concentration at the time of inoculation and produced more hydrogen gas after the same period of growth as the first two batches (68–69 h).

To further investigate the applicability of the thermodynamic framework modified for phototrophic growth, literature-reported experimental data for photoheterotrophic growths were analyzed and converted to the common format of growth yield values. The calculations were done following the thermodynamic framework (see section “Materials and Methods”), with modification regarding the varying electron donors and acceptors for specific cases in the literature. The assembled and analyzed literature data is presented in Table 3. Detailed calculations of predicted thermodynamic growth yields can be found in Supplementary Excel Spreadsheet. Unfortunately, a significant portion of published literature data was incomplete and found to be unusable for this particular study, thus omitted for thermodynamic validation. For instance, many experiments were conducted in nutrient-rich media with the addition of either yeast extract, vegetable stocks, or multiple defined carbon sources (acetate, butyrate, etc.). These variations significantly skew the calculations and resulting data do not match the theoretical predictions. Some studies did not provide a photon-saturated environment for their growth experiment (flux of less than 60 W × m^–2^) or used IR (850–870 nm) light, leading to the somewhat lower reported yields compared to the predictions from theoretical calculations. Finally, the majority of the growth physiology studies do not include spectrophotometer calibration or conversion of Klett units to the dry weight of the cell biomass, resulting in a unusable set of data for growth yield estimations. That was specifically the case for most of the studies describing anaerobic phototrophic N_2_-fixing bacterial growth. As a result, the experimental observation we report here (Table 2) is the only source of anaerobic phototrophic N_2_-fixing growth data.

**TABLE 3:** A compilation of experimental data from literature resources describing growth characteristics of photoheterotrophically grown bacteria.

**Source**	**Organism, growth conditions and nitrogen source**	**Substrate, S**	**Observed**		**Predicted**	
			**Biomass (X) yield,*Y*_*X*/*S*_, mol/mol**	**Hydrogen yield, *Y*_*H*_2_/*S*_, mol/mol**	**Biomass (X) yield,*Y*_*X*/*S*_, mol/mol**	**Hydrogen yield, *Y*_*H*_2_/*S*_, mol/mol**
(McKinlay and Harwood, 2010)	PNSB^1^, batch, 30°C,NH_4_^+^	Acetate	0.31^2^	0.41	0.238	1.6
(Adessi et al., 2012b)	PNSB, 50L PBR, 30°C, pH 6.9, NH_4_^+^	Malate	–	1.1	–	1.34
(Biebl and Pfennig, 1978)	Green sulfur bacteria, batch, 28°C, pH 7.3, NH_4_^+^	Varying	0.3^3^	–	0.45^3^	–
(Adessi et al., 2012a)	PNSB, batch, 30°C, NH_4_^+^	Acetate	–	4.3^4^	–	6.75^4^
(Obeid et al., 2009)	PNSB, 1L PBR, 30°C, NH_4_^+^/glutamate	Lactate, Malate	0.62^5^	2.61^5^	0.39^5^	2.09^5^
(Getha et al., 1998)	PNSB, batch, 30°C, pH (6.5 to 4.5), NH_4_^+^	Sago-starch-processing wastewater	0.24–0.83	–	0.94^6^	–
(Fujii et al., 1993)	PNSB, batch, 30°C, 101.325 kPa, glutamate	Levulinic acid	1.1	1.25–1.4	0.73	3.2
(Barbosa et al., 2001)	PNSB, batch, 30°C, glutamate	Acetate	0.236	0.48	0.238	1.6
(Hu et al., 2018)	PNSB, batch (continuous discharge of H_2_), 30°C, 101.325 kPa, final pH (7.3 for acetate, 6.5 for butyrate, 6.4 for lactate, 9.1 for malate), glutamate	Lactate	–	1.64	–	2.09
		Malate	–	2.35	–	1.34
		Butyrate	–	4.6	–	3.63
		Acetate	–	1.59	–	1.6
(Vasiliadou et al., 2018)	PNSB, batch, IR light, 25°C, 101.325 kPa, pH 6.8, glutamate	Malic acid	0.34	0.68	0.37	1.34
		Acetic acid	0.2	0.52	0.238	1.6
		Butyric acid	0.55	1.07	0.59	3.63
	PNSB, batch, IR light, 25°C, 101.325 kPa, pH 6.8,N_2_	Malic acid	0.24	–	0.254	–
(Padovani et al., 2016)	PNSB, semi-continuous PBR, 30°C, 101.325 kPa, pH 7, glutamate	Acetate	–	1.6	–	1.6
	PNSB, fed-batch PBR, 30°C, 101.325 kPa, pH 7, glutamate		–	1.42	–	
	PNSB, batch PBR, 30°C, 101.325 kPa, pH 7, glutamate		–	0.85	–	
(Nath et al., 2005)	PNSB, batch, 37°C, 101.325 kPa, pH 6.8, glutamate	Acetate	–	1.5–1.72^7^	–	1.6

*All cases described bacteria growing phototrophically unless otherwise stated (ex. IR light). “Observed” stands for the data reported in the literature, while “Predicted” illustrates calculated theoretical yields with the given parameters in the corresponding literature source. ^1^PNSB – purple non-sulfur bacteria; ^2^original data reported for biomass composition varying from C_5_H_7_O_2_N, and conversion was needed to bring to the same format; ^3^Yield is calculated per electron donor consumed (sulfide); ^4^Yield is calculated per biomass produced; ^5^Yield is calculated per lactate consumed; ^6^Predicted yield is calculated using a generic wastewater formula C_10_H_19_O_3_N (Tchobanoglous et al., 2014) and results reported in grams of biomass/gram of wastewater COD or sugar content; ^7^Hydrogen gas production reported at 25°C and 1 atm (101.325kPa).*

As can be seen from the data collected in Table 3 and statistical analysis in Table 4, some reported growth yields fall within a range predicted by the thermodynamic model plus or minus 20%. The biggest sources of inconsistencies are:

**TABLE 4:** The correlation between thermodynamically predicted and observed biomass yield based on the literature-available data presented in Table 3.

**Source**	**Biomass (X) yield,**		**Relative**
	***Y*_*X*/*S*_, mol/mol**		**error, %**
	**Observed**	**Predicted**	
McKinlay and Harwood, 2010	0.31	0.238	23.22
Biebl and Pfennig, 1978	0.3	0.453	51
Obeid et al., 2009	0.62	0.395	36.3
Getha et al., 1998	0.83	0.946	13.97
Fujii et al., 1993	1.1	0.73	33.64
Barbosa et al., 2001	0.236	0.238	0.85
Vasiliadou et al., 2018	0.34	0.37	8.82
Vasiliadou et al., 2018	0.2	0.238	19
Vasiliadou et al., 2018	0.55	0.59	7.27
Vasiliadou et al., 2018	0.24	0.254	5.83
Mean Average Absolute Error (MAE)			0.106

(1) Use of batch cultivation systems with a non-continuous discharge of accumulated hydrogen gas;
(2) Use of glutamate, that is claimed to be a nitrogen source, but can be catabolized as a carbon source as well (Hutner, 1950);
(3) Lack of a study-specific cell biomass composition formula and use of a C_5_H_7_O_2_N generic formula (MW = 113 g × mol^–1^) instead can lead to discrepancies.
(4) The energy of the red-light photon (870 nm) was used for theoretical calculations, which is characteristic for bacteriochlorophyll *a* associated with RC in PNSB. However, some species of PNSB have bacteriochlorophyll *b* associated with RC and absorb 960 nm photons (Woodbury and Allen, 1995; Hoff and Deisenhofer, 1997), while green sulfur bacteria have bacteriochlorophyll in RC structure that absorbs 900 nm photons (Hauska et al., 2001).

Overall, data that best matched the theoretical predictions described a light-saturated environment for cultivation with defined media composition and continuous discharge of the produced hydrogen (where H_2_ production data was available or possible to calculate) (Sasaki et al., 1980; Barbosa et al., 2001; Nath et al., 2005; Padovani et al., 2016; Hu et al., 2018). Since majority of the literature-reported experimental set-ups were not continuously discharging the collected hydrogen gas, correlation between predicted and observed hydrogen gas yields was not as good as for the biomass yields (Table 5).

**TABLE 5:** The correlation between thermodynamically predicted and observed hydrogen gas yield based on the literature-available data presented in Table 3.

**Source**	**Hydrogen yield,**		**Relative**
	***Y*_*H*_2_/*S*_, mol/mol**		**error, %**
	**Observed**	**Predicted**	
McKinlay and Harwood, 2010	0.41	1.6	290.24
Adessi et al., 2012b	1.1	1.34	21.82
Adessi et al., 2012a	4.34	6.754	55.62
Obeid et al., 2009	2.615	2.095	19.88
Fujii et al., 1993	1.33	3.2	140.6
Barbosa et al., 2001	0.48	1.6	233.33
Hu et al., 2018	1.64	2.09	27.44
Hu et al., 2018	2.35	1.34	42.98
Hu et al., 2018	4.6	3.63	21.09
Hu et al., 2018	1.59	1.6	0.63
Vasiliadou et al., 2018	0.68	1.34	97.06
Vasiliadou et al., 2018	0.52	1.6	207.7
Vasiliadou et al., 2018	1.07	3.63	239.25
Padovani et al., 2016	1.6	1.6	0
Nath et al., 2005	1.6	1.6	0
Mean AverageAbsolute Error (MAE)			0.94

An interesting observation was made regarding studies concerned with hydrogen production: a desire to increase capacity for biological hydrogen production is not met with proper engineering designs of the photobioreactors or a choice of a microorganism. It is well established that purple non-sulfur bacteria with functional uptake hydrogenases (most of the wild types) will re-uptake its own produced hydrogen, especially if there is even a trace amount of oxygen present in the media (Kelley et al., 1977; Meyer et al., 1978). Surprisingly, there are many studies with a focus on biohydrogen production from low energetic value substrates (ex. acetate) and a persistent idea that hydrogen yields can be improved in this case. That goal is unattainable, since thermodynamic calculations predict maximum theoretical hydrogen yield from acetate as one of the lowest among any other organic substrates: 1.6 mol H_2_/mol acetate (Supplementary Material and correspondent data in the Table 3). Hydrogen yields from substrates such as glucose, butyrate or even municipal wastewater are much higher (3, 4, and 8.2 mol/mol, respectively) (Table 3). Of course, multiple different substrates can be mixed to achieve higher yields, but substrate competition should not be disregarded in this case. Genetic manipulations can also be made to the microbial catalysts to increase the product yields, but it seems like the natural potential is not fully exploited either.

This example of using a simple thermodynamic framework to analyze biohydrogen potential and routes to increase its biological yield is just one in many. Our experimental observation of purple non-sulfur bacterial growth demonstrates a feasible approach to use diazotrophic organisms for biotechnological purposes. However, more studies with N_2_-fixing microorganisms are needed to fill the gap in modeling data repository and help develop better prediction tools for microbial growth. With the rising need for a sustainable production of nitrogen biofertilizers (Dogutan and Nocera, 2019), studies with diazotrophic microorganisms should become more and more widespread.

## Conclusion

The presented modification to the thermodynamic framework includes photon-associated energy for estimation of bacterial growth yield and product formation. The yield metrics predicted by the improved modified framework have been successfully tested on the experimental and literature-available data. However, due to the scarcity of the available literature data on phototrophic and diazotrophic bacterial growth, further validation is necessary. The improved thermodynamic framework presented in this research, originally established by McCarty (McCarty, 1965, 2007; Rittmann and McCarty, 2001), can have useful applications for any biotechnological applications where preliminary planning for the choice of microbial catalyst is a must. Application of the thermodynamic framework to the analysis of phototrophic microbial growth will allow to predict substrate conversion stoichiometries in broader environmental niches and assess the possibility for the development of novel biotechnological applications in light-induced biological systems. This is especially important for biotechnological applications like biohydrogen production (Bolatkhan et al., 2019), phototrophic production of polyhydroxyalkanoates (Higuchi-Takeuchi et al., 2016), use of cyanobacteria as cell factories (Li and Liao, 2013) and photobiological wastewater treatment (Sawayama et al., 1999). The ability to predict and evaluate growth parameters in otherwise energy scarce environments (photoautotrophic lifestyles) might point to the new areas of basic research with the isolation of novel energy-efficient microbes.

## Data Availability Statement

All datasets generated for this study are included in the article/Supplementary Material.

## Author Contributions

AD conceived the study, designed and performed the experiments, analyzed the data. AD wrote the manuscript in consultation and support of LS. Both authors contributed to the article and approved the submitted version.

## Conflict of Interest

The authors declare that the research was conducted in the absence of any commercial or financial relationships that could be construed as a potential conflict of interest.
