# Supplementary material for: An Experimentally Evaluated Thermodynamic Approach to Estimate Growth of Photoheterotrophic Purple Non-sulfur Bacteria
Source: Front Microbiol. 2020 Sep 3;11:540378. doi: 10.3389/fmicb.2020.540378 (PMC7494753; doi:10.3389/fmicb.2020.540378)
Supplement: Supplementary file 1 [file Data_Sheet_1.docx]

Supplementary Material for “An Experimentally Evaluated Thermodynamic Approach to Estimate Growth of Photoheterotrophic Purple Non-sulfur Bacteria” by Anna Doloman and Lance C. Seefeldt

# Supplementary Data

Below are the calculations of stoichiometric biomass and hydrogen production for purple non-sulfur bacteria grown in minimal defined media with N_2_ as a source of nitrogen and acetate as a carbon source. The thermodynamic assumptions and equations for electron donor and acceptor half-reactions come from literature sources (Thauer et al., 1977; Rittmann and McCarty, 2001; Tchobanoglous et al., 2014), while photon-associated energy is calculated with a novel approach proposed in the current study (see Eq.1 in the main text). Detailed calculations can be also found in the Supplementary Excel spreadsheet, associated with the manuscript. Table 1 (from the main text) provides the summary of half-reactions for electron acceptor and electron donor, needed to calculate the Gibbs free energy available for the microbial growth.

Based on the original framework and assumptions proposed by McCarty (Rittmann and McCarty, 2001), the obtained energy for the biological system, $\boldsymbol{\Delta}G_{R}$, calculated from energy associated with half-reactions and photons in TableS1, is distributed between catabolic and anabolic cellular processes: $\begin{aligned} f_{e}\times K \times\left( \Delta G_{R} \right)+f_{s}\times(\Delta G_{s})= 0\#\left( S1 \right) \end{aligned}$

While$\begin{aligned} f_{e}+f_{s}=1 \#\left( S2 \right) \end{aligned}$

Where, $\Delta G_{r}$is the energy released from oxidation-reduction reactions; $f_{e}$ is e^-^ mole of substrate oxidized per e^-^ mole of substrate used (fraction of electron donor used for energy, to fuel catabolic cellular processes); $f_{s}$ is e^-^ mole of substrate used for cell synthesis per e^-^ mole of substrate used (fraction of electron donor used for cell synthesis, anabolic cellular processes); $\Delta G_{s}$is the energy used for cell growth. Calculations for 𝛥G_s_ can be done using the following equation:

$$\begin{aligned} \Delta G_{s}= \frac{\Delta G_{P}}{K^{m}}+\Delta G_{C}+ \frac{\Delta G_{N}}{K} \#\left( S3 \right) \end{aligned}$$

Where: $\Delta G_{N}$is 16.36 kJ/eeq (free energy per e^-^ eq of cells to reduce dinitrogen to ammonia, and will be different if the cell receives a different form of nitrogen (NO_3_^-^, NO_2_^-^ or NH_3_/NH_4_^-^) (Rittmann and McCarty, 2001)); $\Delta G_{C}$ is 31.41 kJ/eeq of cell (energy needed to convert 1 e^-^ eq of pyruvate intermediate into 1 e^-^ eq of cells (Rittmann and McCarty, 2001)); $K$ is 0.55 (fraction of energy transfer captured); m = "+1" if $\Delta G_{P}$ is positive, and "-1" if energy is produced; $\Delta G_{p}$is free energy to convert 1 e^-^ eq of carbon source to the pyruvate intermediate. By solving the system of equations (Eq. S1 – S2), one can find all the relevant coefficients to estimate microbial growth rates. Table S1 provides a summary of the calculations. Subsequently, the overall stoichiometry of biological reaction can then be defined with:

$$\begin{aligned} R= f_{e}\times R_{a}+ f_{s}\times R_{cs}- R_{d} \#\left( S4 \right) \end{aligned}$$

Where: $R$ is an overall balanced reaction; $R_{a}$ is a half-reaction for electron acceptor; $R_{cs}$ is a half-reaction for the synthesis of cell tissue; $R_{d}$is a half-reaction for electron donor. Values for $f_{e}$ and $f_{s}$ were calculated from Eq. S1-S2 (Table S1). .$\begin{aligned} 0.507\times R_{a}=0.507\times\left( \frac{1}{8} N_{2}+\frac{5}{4} H^{+}+e^{-}\to\frac{1}{4} {NH}_{4}^{+}+\frac{1}{8} H_{2} \right)= \\ =0.063 N_{2}+0.634 H^{+}+{0.507 e}^{-}\to\\ 0.1268 {NH}_{4}^{+}+0.0634 H_{2} \#\left( S4.1 \right) \end{aligned}$

$$\begin{aligned} 0.493\times R_{cs}=0.493\times\left( \frac{5}{23} CO_{2}+\frac{1}{46} N_{2}+{+H^{+}+e}^{-}\to\frac{1}{23} C_{5}H_{7}O_{2}N+\frac{8}{23} H_{2}O \right)= \\ 0.107 CO_{2}+0.0107 N_{2}+{+0.493 H^{+}+0.493 e}^{-}\to\\ \to0.0214 C_{5}H_{7}O_{2}N+0.1714 H_{2}O \#\left( S4.2 \right) \end{aligned}$$

$$\begin{aligned} -R_{d}= \frac{1}{8}CH_{3}COO^{-}+\frac{3}{8} H_{2}O\to\frac{1}{8} CO_{2}+ \frac{1}{8} HCO_{3}^{-}+ H^{+}+ e^{-} \#\left( S4.3 \right) \end{aligned}$$

$$\begin{aligned} R=0.125 CH_{3}COO^{-}+0.0741 N_{2}+0.127H^{+}+0.2036 H_{2}O \to\\ \to0.0214 C_{5}H_{7}O_{2}N+0.018 CO_{2}+0.125 HCO_{3}^{-}+0.127 {NH}_{4}^{+}+0.063 H_{2} \#\left( S4.4 \right) \end{aligned}$$

Where: $C_{5}H_{7}O_{2}N$ is a general formula describing bacterial biomass composition (MW = 113 g/mol) (Rittmann and McCarty, 2001).

The resulting stoichiometrically-balanced equation (R) for the anaerobic photoheterotrophic growth of purple non-sulfur bacteria growing with N_2_ as a source of nitrogen and acetate as a carbon source can be used to calculate growth yields $\left( \boldsymbol{Y}_{\boldsymbol{X}/\boldsymbol{S}} \right)$ and product yields $\left( \boldsymbol{Y}_{\boldsymbol{P}/\boldsymbol{S}} \right)$.

Cells produced: 0.0214 mol × 113 g VSS/mol = 2.42 g VSS cells

Acetate used: 0.125 mol × 59 g/mol = 7.375 g acetate

Hydrogen produced: 0.063 mol × 23.455 L/mol = 1.49 L

Yield of biomass on acetate, $\boldsymbol{Y}_{\boldsymbol{X}/\boldsymbol{Acetate}}$: 2.42 g VSS cells / 7.375 g acetate = 0.328 g cells/ g acetate

Yield of hydrogen on acetate, $\boldsymbol{Y}_{{\boldsymbol{H}_{\boldsymbol{2}}}/\boldsymbol{Acetate}}$**:** 1.49 L H_2_ / 7.375 g acetate = 0.2 L H_2_ / g acetate.

# Supplementary Tables

**Table S1.** Parameters estimation.

| The energy available for cellular needs, ${kJ}/{mol eeq}$ | $K\times\left( \Delta G_{R} \right)=0.55\times\left( -133.96 \right)= -73.68$ |
| --- | --- |
| Energy to convert 1 e^-^ eq of carbon source to the pyruvate intermediate,${kJ}/{mol eeq}$ | $\frac{1}{8}CH_{3}COO^{-}+\frac{3}{8} H_{2}O\to\frac{1}{8} CO_{2}+ \frac{1}{8} HCO_{3}^{-}+ H^{+}+ e^{-}$  $\left( \Delta G^{0}=-27.68 {kJ}/{mol eeq} \right)$  $\frac{1}{5} {CO}_{2}+\frac{1}{10} HCO_{3}^{-} + H^{+}+ e^{-}\to\frac{1}{10}CH_{3}CO{COO}^{-}+\frac{2}{5}H_{2}O$  $\left( \Delta G^{0}=35.78 {kJ}/{mol eeq} \right)$  $\Delta G_{p}=(-27.68)+35.78=8.1$ |
| Energy for cell synthesis, ${kJ}/{mol eeq}$ | $\Delta G_{s}=\frac{8.1}{{0.55}^{+1}}+31.41+ \frac{16.36}{0.55}=75.88$ |
| The fraction of energy for catabolic cellular needs | $f_{e}=0.507$ |
| The fraction of energy for anabolic cellular needs | $f_{s}=1- 0.507=0.493$ |

# Supplementary Figures

Figure S1. Spectrum of the 60 W incandescent light used for the photobioreactor growth experiment.

Figure S2. Spectrum of the 17 W fluorescent lamp used for the photobioreactor growth experiment.

# References

Rittmann, B. E., and McCarty, P. L. (2001). *Environmental biotechnology : principles and applications*. New York: McGraw-Hill Higher Education.

Tchobanoglous, G., Burton, F. L., Stensel, H. D., and (Boston), M. & E. (2014). *Wastewater engineering : treatment and resource recovery*. New York: McGraw-Hill Higher Education.

Thauer, R. K., Jungermann, K., and Decker, K. (1977). Energy conservation in chemotrophic anaerobic bacteria. *Bacteriol. Rev.* 41, 100–180. Available at: https://www.ncbi.nlm.nih.gov/pubmed/860983.
